# Variability in depression prevalence in early rheumatoid arthritis: a comparison of the CES-D and HAD-D Scales

**DOI:** 10.1186/1471-2474-10-18

**Published:** 2009-02-07

**Authors:** Tanya Covic, Julie F Pallant, Alan Tennant, Sally Cox, Paul Emery, Philip G Conaghan

**Affiliations:** 1 School of Psychology, University of Western Sydney, Locked Bag 1797, Penrith South DC 1797, NSW, Australia; 2 School of Rural Health, University of Melbourne, 49 Graham St, Shepparton, VIC, Australia; 3 Department of Rehabilitation Medicine, Faculty of Medicine and Health, University of Leeds, D Floor, Martin Wing, Leeds General Infirmary, Leeds, LS1 3EX, UK; 4 Section of Musculoskeletal Disease, Leeds Institute of Molecular Medicine, University of Leeds, 2nd floor Chapel Allerton Hospital, Chapeltown Road, Leeds, LS7 4SA, UK

## Abstract

**Background:**

Depression is common in rheumatoid arthritis (RA), however reported prevalence varies considerably. Two frequently used instruments to identify depression are the Center for Epidemiological Studies Depression (CES-D) scale, and the Hospital Anxiety and Depression Scale (HADS). The objectives of this study were to test if the CES-D and HADS-D (a) satisfy current modern psychometric standards for unidimensional measurement in an early RA sample; (b) measure the same construct (i.e. depression); and (c) identify similar levels of depression.

**Methods:**

Data from the two scales completed by patients with early RA were fitted to the Rasch measurement model to show that (a) each scale satisfies the criteria of fit to the model, including strict unidimensionality; (b) that the scales can be co-calibrated onto a single underlying continuum of depression and to (c) examine the location of the cut points on the underlying continuum as indication of the prevalence of depression.

**Results:**

Ninety-two patients with early RA (62% female; mean age = 56.3, SD = 13.7) gave 141 sets of paired CES-D and HAD-D data. Fit of the data from the CES-D was found to be poor, and the scale had to be reduced to 13 items to satisfy Rasch measurement criteria whereas the HADS-D met model expectations from the outset. The 20 items combined (CES-D13 and HADS-D) satisfied Rasch model expectations. The CES-D gave a much higher prevalence of depression than the HADS-D.

**Conclusion:**

The CES-D in its present form is unsuitable for use in patients with early RA, and needs to be reduced to a 13-item scale. The HADS-D is valid for early RA and the two scales measure the same underlying construct but their cut points lead to different estimates of the level of depression. Revised cut points on the CES-D13 provide comparative prevalence rates.

## Background

Depression is common in rheumatoid arthritis (RA) with prevalence rates ranging from 13 to 20% [1,2] based on psychiatric assessment and clinical diagnosis of depression, and considerably higher when based on self-report assessment.[3] The co-morbidity of depression in RA exceeds the rates of depression reported in the general community (2–4%) and primary care (5–10%).[4] Depression in RA is closely associated with pain, work disability, health services utilisation, poor adherence to treatment and suicide.[5]

Regular mood assessment by rheumatology clinical staff may serve to improve awareness and early identification of depression,[5] and thus timely identification and treatment of depression in RA is critical to overall clinical management.[6] While not substituting for a psychiatric clinical assessment, the use of self-report scales may be a feasible option in rheumatology settings to identify patients at risk of depression. Regular screening, and early intervention or appropriate referral, where necessary, would provide a psychological 'window of opportunity' [7] akin to that recommended in relation to clinical treatment of early RA.

While a number of screening instruments for depression are available, two of the most commonly used scales are the Hospital Anxiety and Depression Scale (HADS) [8] and the Center for Epidemiological Studies Depression (CES-D) scale.[9] The HADS was developed for use in hospital settings, with items chosen to reduce contamination with somatic or disease related symptoms. The CES-D was originally designed for use with the general population [9] but has been found to be valid and reliable in the identification of individuals at high risk of developing a major depression in clinical populations including RA.[10] Both scales have been used with RA populations [1,11-17] and have been subjected to psychometric assessment of their reliability and validity including exploratory and confirmatory factor analyses.[18,19] In recent years these traditional psychometric methods have been supplemented by modern psychometric approaches such as the Rasch measurement model. [20-22] A number of studies have used Rasch analysis to assess the CES-D, including a comparison of stroke versus primary-care patients;[23] investigation of phone interview versus mail administration;[24] and the development of a short-form CES-D.[25] Only one study has tested CES-D in an RA population [26] and failed to find support for the original 20-item version. A modified 13-item version of the scale was found to provide a more satisfactory fit to the Rasch model. Rasch analysis of the HADS has found support for the psychometric properties of the scale in a musculoskeletal sample [27] and a cancer population [28] but has not been assessed in an RA population.

Given that CES-D and HADS are two of the more commonly used depression scales, they serve as a good starting point to assess whether the case definition of depression scales is a potential source of the variation in prevalence of depression reported in the RA literature. This is important to establish, as it is not possible to screen all RA patients with a clinical diagnostic interview, due to cost and access limitations. A valid self-report scale is therefore essential if screening for depression is to become routine, as recommended, [5,29] and be reliably predictive of depression levels. Rheumatologists should be confident that the screening instruments available to them identify the same level of depression or, if not, then there should be a clear understanding of why this is the case. Therefore, the aims of this study were to: (a) assess the modern psychometric properties of the CES-D and HADS in a cohort of early RA patients; (b) to co-calibrate the items from both scales on to a single metric to determine if they do measure the same construct, and (c) to compare their respective cut points for depression to determine if the variation of reported prevalence may be due to variability of scale-specific case definition.

## Methods

### Study Participants

Data from the CES-D and HADS items were obtained from patients attending the Yorkshire Early Arthritis Register clinic in Leeds, UK. The participants met the RA diagnosis according to the 1987 American College of Rheumatology criteria.[30] Ninety-two early-RA participants (62% female; mean age = 56.3, SD = 13.7) were assessed twice within a six month period. The majority of patients (66%) had RA duration of less than two years, the remaining around three years. Patients received standard disease modifying anti-rheumatic drugs (DMARD) therapy according to a predetermined local protocol. Ethical approval was granted by the relevant Research Ethics Committee, under a programme of work for the analysis of common musculoskeletal outcome measures.

### Measures

CES-D is a 20-item scale designed to measure depressive symptoms experienced in the past week.[9] Responses range from 0 to 3 where 0 = *Rarely or none of the time (less than 1 day)*; 1 = *Some or a little of the time (1–2 days)*; 2 = *Occasionally or a moderate amount of the time (3–4 days)*; and 3 = *Most or all of the time (5–7 days)*. Four of the items are positively worded and are reverse-scored before adding all items to give a total CES-D score. Scores range from 0 to 60, with a cut-off of 16 indicative of probable clinical depression.[9] A higher cut-off of 19 has been suggested in the RA population and other primary care clinical populations as a more optimal scale sensitivity point that avoids 'false-positive' identification of depression cases. [31]

HADS is a 14-item scale consisting of two 7-item subscales measuring depression and anxiety on a 4-point response scale ('0' indicating absence of symptoms, to '3' indicating maximum symptoms). [8] Subscale scores range from 0 to 21, with higher scores indicating higher levels of depression and/or anxiety. Scores between 0 and 7 represent *no *case; 8 to 10 indicates *possible *case and 11 to 21 suggest a *probable *case of depression/anxiety.[8] In this study only the HADS-Depression subscale (HADS-D) was used.

### Rasch Analysis

Rasch analysis takes its name from the Danish mathematician Georg Rasch.[20]. He introduced a model which specifies what is needed to construct interval level measurement from ordinal scales (e.g. those typically derived from questionnaires), and consequently it acts as a template against which the data can be evaluated. There are versions of the model for scales which have dichotomous items, and polytomous items. [20,21,32] The process of Rasch analysis is an iterative procedure which assesses fit of data to the model, a number of measurement attributes, as well as the assumptions which underpin the model. [27,33] In RUMM2020,[34] the Rasch analysis package used in this study, three overall fit statistics to assess fit of data to the model are considered; two of which are item-person interaction statistics transformed to approximate a z-score, representing a standardized normal distribution. Therefore when the items and persons fit the model the mean is approximately zero with a standard deviation of 1. A third summary fit statistic is an item-trait interaction statistic reported as a Chi-Square. A significant Chi-Square indicates that there is deviation from model expectations and is indicative of poor fit. In addition to these overall summary fit statistics, individual person and item fit statistics are presented, both as residuals and as a chi square statistic. In the former case, residuals between ± 2.5 are deemed to indicate adequate fit to the model. A chi-square test is also available for each item and again good fit to model expectations would be indicated by a non-significant value. To take account of multiple testing, Bonferroni corrections are applied to adjust the Chi-Square p value.[35]

Other aspects of Rasch analysis are concerned with testing model assumptions, such as local dependency and unidimensionality, and with the investigation of other attributes such as appropriate category response structure (for polytomous items) and for item bias, or Differential Item Functioning (DIF).[36] Local dependency is identified through correlations in the residuals, typically above 0.3. Unidimensionality is an assumption of the Rasch model and within RUMM2020 this is tested by a comparison of two independent estimates for the same person, based upon different sets of items identified by the principal component analysis of the residuals [37]. Less than 5% of the tests should be significantly different (or the lower bound of the binomial confidence interval should overlap 5%) for the scale to be considered unidimensional

For polytomous items, the assumption of the rating scale version of the model is that the distances between thresholds (the transition point between response categories) must be equal across all items [21] otherwise an unrestricted, or partial credit version of the model is used.[32] Thresholds must also show an increase consistent with the underlying trait (that is, an increase in the response option is associated with an increase in the underlying trait), else they are considered 'disordered' which can affect fit statistics. In these circumstances categories are often collapsed to improve fit.

Finally a measure of reliability is provided in the form of a Person Separation Index, which can be interpreted as a Cronbach's alpha. Although the number of items in the scale need to be taken into account, typically values of 0.7 and above are considered suitable for group use, and 0.85 and above for individual use.[38] This is closely linked to the targeting of the scale as it differentiates the number of statistically distinct groups of respondents that can be identified on the trait.[39] Given these procedures, adequate fit to the Rasch model, confirmation of unidimensionality and freedom from item bias (DIF) support the internal construct validity of the scale. [40,41]

## Results

From the 92 participants at time 1 and 2, 141 sets of paired CES-D and HAD-D data were usable for Rasch analyses. In both cases, the unrestricted or partial credit version of the model was found to be most appropriate.

### Rasch Analysis of CES-D

CES-D scores ranged from 0 to 55 with a mean of 16.7 (SD = 11.3). Initial inspection of the scale showed poor overall fit to the Rasch model as indicated by a significant item trait interaction (*χ*^2 ^= 314.148, df = 40, p < 0.001) and item fit residual values outside the acceptable range (mean = -0.139, SD = 2.588).

Ten of the items were found to have disordered thresholds, suggesting problems with the 4-point response format used for the scale. It was therefore decided to rescore all items by merging the two middle categories ('*some or a little of the time*' and '*occasionally or a moderate amount of the time*') thus reducing the scoring to a 3-point format from 0123 to 0112, and making the overall score range 0 to 40.

Following this, seven misfitting items were identified with significant chi-square probability values or high positive or high negative residual values (± 2.5). Items were removed one at the time, based on the magnitude of the significant chi-square probability value and positive or negative residual values, with the overall model fit and individual item statistics checked after each step, until all remaining items were shown to fit model expectations (Table 1). The seven removed items were items: 2, 4, 8, 11, 12, 16 & 18.

**Table 1:** Final fit of the CES-D items to the Rasch model

**CESD** **Item**	**CESD Item Name**	**Location**	**SE**	**Fit Res**	**DF**	**ChiSq.**	**DF**	**Prob.**
1	Bothered	-0.617	0.197	0.175	97.57	1.468	2	0.479
3	Blues	0.100	0.211	-2.028	96.67	7.553	2	0.023
5	Concentrate	-0.909	0.192	0.380	96.67	0.802	2	0.669
6	Depressed	-0.486	0.210	-1.973	95.76	6.573	2	0.037
7	Effort	-1.920	0.183	1.025	94.86	6.853	2	0.032
9	Failure	0.143	0.208	-0.279	96.67	0.196	2	0.907
10	Tearful	-0.394	0.205	-1.853	94.86	2.659	2	0.264
13	Talked less	0.065	0.212	0.969	94.86	0.192	2	0.908
14	Lonely	-0.071	0.209	-1.127	93.95	2.031	2	0.362
15	Others unfriendly	0.899	0.267	0.714	94.86	6.561	2	0.037
17	Crying spells	0.211	0.216	-0.434	95.76	1.261	2	0.532
19	Others dislike	4.539	0.321	-0.747	95.76	0.659	2	0.719
20	Not get going	-1.560	0.198	-0.061	95.76	0.290	2	0.865

The final solution, retaining 13 items, showed overall fit to the model (Table 2). The person separation reliability, which is equivalent to Cronbach's alpha was found to be high (PSI = 0.916), making it suitable for individual use. The items of the CES-D13 scale were assessed for differential item functioning (DIF) across time (1 and 2), gender (male/female), and age (3 groups: ≤ 53 yrs; 54–65 yrs; 66+ yrs) using a Bonferroni-adjusted alpha value. The only significant DIF was found on Item 3 ('*I felt that I could not shake off the blues even with help from my family or friends*') for gender, with females more likely to endorse it than males (p = 0.0005). As overall fit to the model was achieved it was decided not to remove this item and to retain it for further investigation.

**Table 2:** Overall Rasch model fit statistics

**Scale**	**χ^2^**	**p**	**Item** **Fit Residual mean (SD)**	**Person** **Fit Residual mean (SD)**
20-item CES-D	314.15	0.00	-0.14 (2.59)	-0.10 (1.03)
13-item CES-D	37.10	0.07	-0.40 (1.08)	-0.36 (1.11)
7-item HADS-D	16.78	0.27	-0.23 (0.61)	-0.29 (0.77)
CES-D13+HADS-D	58.37	0.03	-0.14 (0.85)	-0.27 (1.16)

To test the unidimensionality of the CES-D13 scale, a Principal Components Analysis of the residuals was conducted to identify the two most divergent subsets of items as indicated by positive and negative loading items on the first component extracted. Comparison of the person estimates generated from these two subsets indicated that eight (7.41%) of the 108 t-tests showed significant differences in the estimates generated, which was non-significant when a 95% confidence interval from a Binomial distribution was applied. This supports the unidimensionality of the CES-D13 scale.

### Rasch Analysis of HADS – Depression (HADS-D)

HADS-Depression scores ranged from 0 to 20 with a mean of 7.4 (SD = 4.2). The seven items of the HADS-D scale showed satisfactory fit to the Rasch (Table 2). All items showed ordered thresholds, indicating appropriate use of the response format. There was no evidence of item or person misfit. No differential item functioning was found for time, gender or age. Principal component analysis of the residuals identified two sets of contrasting items from which individual person estimates were obtained. Only six (5.13%) of the t-tests showed significant differences in the estimates generated from each subset of items, which was non-significant when a 95% confidence interval from a Binomial distribution was applied. This supports the unidimensionality of the 7-item HADS-D scale.

### Rasch Analysis of the combined items from the CES-D13 and HADS-D

Data from the CES-D13 and HADS-D were combined and showed satisfactory fit to the Rasch model (Table 2). Although some marginal threshold disturbance was evident in one CES-D13 and three HADS-D items, these were left unaltered as the overall fit of the model was satisfactory. One CES-D13 item (item 3) showed significant uniform DIF, with higher endorsement by females than males (p = 0.0006). Given overall fit to the model, the item was retained. Principal component analysis of the residuals revealed a set of positive and negative loading items, a mixture from both scales. Comparing estimates based upon these subsets, 11 (7.86%) of the 140 t-tests showed significant differences in the estimates generated, which was non-significant when a 95% confidence interval from a Binomial distribution was applied. This supports the unidimensionality of the combined CES-D13 and HADS-D items demonstrating that both scales measure the same underlying construct.

**Figure 1:**
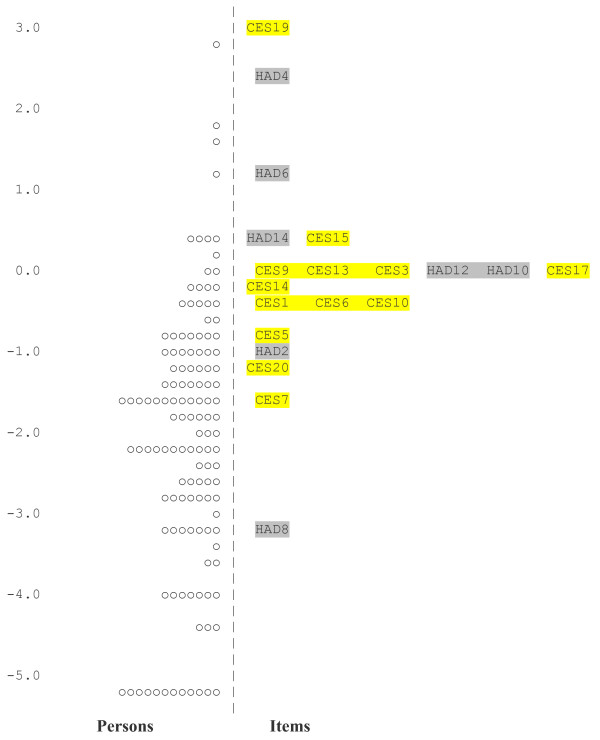
**Item map showing location values for the combined items of the CES-D13 and HADS-D**. The item map shows the relative positioning of the items in terms of their difficulty. CES-D item 19 is the most difficult item to endorse and therefore indicative of the severe end of the levels of depression, whereas HADS-D item 8 is the least difficult item to endorse and therefore indicative of the lowest of depression levels.

Figure 1 shows the person item distribution of the 20 co-calibrated items, suggesting that the HADS-D has a better distribution of items across the range of depression than does the CES-D13. With the exception of item 19, the majority of CES-D13 items are clustered within the centre of the scale. The two scales taken together appear to offer a wider measurement of depression than each scale on their own.

### Comparison of CES-D and HADS-D Case Ascertainment

The difference in levels of depression expressed by items in the CES-D in its various forms and the HADS-D are shown in Table 3. The cut points for the HADS give a prevalence of *possible *and *probable *depression as 22.6% and 9.7% respectively. In contrast the cut-off value (16) to identify depression for the 20-item CES-D scale [9] gives a prevalence of 45.3%, while the RA-specific cut value of 19 [31] gives a prevalence of 35.9%. As such the depression prevalence is much higher for the CES-D than the HADS-D. The equivalent cut points for *possible *and *probable *depression in early RA on the revised CES-D13 would be 9 (possible case – that is the equivalent of an 8 on the HADS-D) and 13 (probable case) respectively, derived from the 13-item scale scored 0112 (i.e. a total score range on 0–26). Based on those cut points, 26.6% of the participants had possible depression and 8.1% probable cases of depression, closely aligned to the rates identified by the HADS-D. Given the prevalence from the HADS-D corresponds most closely with the reported prevalence based on psychiatric interview, this scale would seem most suitable to offer as an 'interim' gold standard for comparing other scales, in the absence of further clinical interviews.

**Table 3:** Depression cut-off scores and case percentages

**Scale**	**Cut-off Score**	**%**
HADS-D	11 (probable case)	9.7
HADS-D	8 (possible case)	22.6
20-ITEM CES-D	19 (RA cut-off)	35.9
20-ITEM CES-D	16 (standard cut-off)	45.3
CES-D13	9 (probable case)	8.1
CES-D13	13 (possible case)	26.6

## Discussion

The aim of this study was to test a number of issues associated with using self-report scales to screen for depressive symptoms in an early RA population. Using a modern psychometric approach, namely Rasch analysis, data from two widely used scales for depression were collected concurrently and initially tested against Rasch model requirements, including unidimensionality. Once these conditions were satisfied, items from the two scales were co-calibrated onto a single measure of depression to demonstrate that they target the same construct, which allowed for comparison of their cut points.

Several significant findings emerged. The 20-item version of the CES-D was found to lack adequate fit to Rasch model expectations, and needed revising to a 13-item scale. Problems with the 20-item version of the CES-D have been identified previously, for example, in a longitudinal RA study.[26] Four (items 4, 8, 12 & 16) of the seven items removed were positively worded and it has been suggested in other studies [25,42] that such items may not measure the same construct as the negatively worded items. The remaining three (2, 11 & 18) removed items relate to somatic features (appetite and sleep) and depressed affect (sadness). Problems with somatic items in CES-D have also been noted in previous studies with RA population. [10,14,43,44] In addition, the response format also needed modification to more accurately reflects participants' responses. Our findings correspond to those reported in relation to the use of CES-D with a more established RA population [26] and suggest that this modified version, CES-D13 may be more clinically useful than the original 20-item CES-D.

Individually, the revised CES-D13 scale and the HADS-D were found to meet Rasch model expectations, and to satisfy strict tests of unidimensionality. It is important to note that unidimensionality is a requirement for summating any set of items [45], even where the scale may consist of related factors such as negative affect and somatic features, as in the case of depression. It is also important to note that unidimensionality is a necessary, but not sufficient, condition for satisfying Rasch model expectations, as the model imposes extra constraints on the data to satisfy the rules for constructing interval scale data [46]. Consequently whether or not measurement of a single construct such as depression can be achieved is an empirical question. Given the pooled item set (CES-D13 + HADS-D) also satisfied Rasch model expectations and unidimensionality tests, this confirms that the two scales do indeed measure the same underlying unidimensional construct.

The variation in reported prevalence of the two scales may be partly explained by the somatic and positive items in the CES-D as well as CES-D being only an indication of depression rather than diagnosis of clinical depression. Furthermore, the lack of unidimensionality in the original CES-D suggests that the 20-item scale measures two different, if related, constructs which may inflate the score and therefore, indication of depression. Both the 20-item scale and the 13-item version of the CES-D displayed at least one item with DIF, and there are issues about the potential bias which may be introduced under such circumstances. Although uniform DIF may be adjusted within the framework of the Rasch measurement model [47], this is not something that can be addressed easily within a routine use of this scale in a clinical setting.

In contrast, the convergence of the HAD-D cut points with the range reported for psychiatric interview would appear to make this scale a useful indicator of a clinical depression and as such a baseline comparator for other scales, as illustrated with the CES-D13 in this study. Indeed, in the absence of clinical diagnostic interviews, the HADS-D can be used as an interim 'gold standard'. Since the modified version of the CES-D contains a reduced response format, further investigation is necessary to confirm its appropriateness, to test-retest the modified scale, and ascertain the positive predictive value of the CES-D (and indeed the HADS-D as well), to determine the scale's utility within a routine clinical setting. Furthermore, given the variability of case ascertainment across the two scales in the current study, further research would appear necessary with other commonly used depression scales (e.g. Beck's Depression Inventory[48]) to see if they too measure the same construct, and to ascertain their comparative cut points.

There are a number of limitations to this study. The sample size is small and is at the viable lower limit for Rasch analysis. This would give a degree of precision of person and item estimate to within just under one logit, which may affect the estimate of sensitivity and specificity. The study population is patients with early RA, and while the findings are consistent to those reported in another cohort of patients with RA [26] further replication is necessary. In the current study no clinical assessment of depression was conducted to provide formal evaluation of cut point sensitivity and specificity of the combined scale, although the prevalence using the HADS-D and the revised CES-D13 is consistent with reported rates for depression in RA using clinical interviews.

The results of this study have a number of implications for research and practice. The current study suggests that variation in prevalence of depression may be a function of the scale used, and many existing scales may give rise to high numbers of false positives in an early RA sample. Where scales are used to identify depression and referral for treatment, the HADS-D appears to provide estimates consistent with clinical diagnostic interviews and thus rheumatologists and others can have some confidence in case ascertainment using this scale. If the CES-D is routinely collected, then the revised 13-item version and modified cut points would provide similar caseness, although further work needs to be done to confirm the internal construct validity of this modified version, as well as criterion validity to confirm sensitivity, specificity and the positive predictive power of the test using these cut points.

Eventually other depression scales, in addition to CES-D and HADS-D, which are shown to measure the same construct can be all calibrated onto a single metric 'item bank' [49] which will pave the way for the development of a computer adaptive testing approach [50] to provide a simple screening tool suitable for use within a rheumatology clinic to allow early identification of depression in RA.

In addition, comparison of scales, such as conducted in this study, provides insight into the variability of the levels of depression detected across studies. It also contributes towards a better understanding of the impact of depression upon, for example, quality of life, when adjusted for the variability of case identification across different screening instruments. In the meantime, the HADS-D can offer the rheumatologist reliable and valid comparative screening for depression in early rheumatoid arthritis. The CESD-13 requires further work concerning reliability and validity of its response structures, but the cut points given above will allow for comparison of existing data sets where the two scales have been used. Clinicians can have confidence that those patients identified as probable cases by the cut points given on either scale will, in existing data sets, be indicative of a possibly clinical depression sufficient for referral and further assessment. For prospective studies, the HADS-D provides the better option of the two scales reviewed.

## Conclusion

The HADS-D met Rasch model expectations and appears to be a useful self-report scale for screening for depression in early RA, as it identifies levels of depression consistent with those reported in other studies using clinical diagnostic interviews. In contrast, the CES-D required modification for use in early RA, and its response structure also necessitated collapsing from a four to a three-category structure. The cut points on the both original CES-D and modified CES-D13 gave much higher levels of possible depression compared to the HADS-D, and may require adjusting, if, for the present time, the HADS-D is considered to be an interim gold-standard. However, the HADS-D and the CES-D13 did successfully co-calibrate on to the same metric, suggesting that they do indeed measure the same construct. Taken together the two scales appear to offer a more comprehensive measure of depression in that their combined width of the depression construct is wider than either scale on their own.

## Competing interests

The authors declare that they have no competing interests.

## Authors' contributions

AT, PGC, TC & JFP conceived of the study; AT, TC & JFP performed the statistical analysis; PGC and PE coordinated data collection; SC conducted data collection, TC, AT and JFP drafted the manuscript. All authors read and approved the final manuscript.

## Pre-publication history

The pre-publication history for this paper can be accessed here:


